# Gaze Behavior in Social Fear Conditioning: An Eye-Tracking Study in Virtual Reality

**DOI:** 10.3389/fpsyg.2020.00035

**Published:** 2020-01-23

**Authors:** Jonas Reichenberger, Michael Pfaller, Andreas Mühlberger

**Affiliations:** Department of Clinical Psychology and Psychotherapy, Institute of Psychology, University of Regensburg, Regensburg, Germany

**Keywords:** social anxiety disorder, social fear conditioning, vigilance-avoidance hypothesis, eye-tracking, virtual reality

## Abstract

The vigilance-avoidance hypothesis of selective attention assumes that socially anxious persons initially direct their attention toward fear-related stimuli and subsequently avoid these social stimuli to reduce emotional distress. New technical developments provide tools to implicit measure overt attention on fear-related stimuli via eye-tracking in ecological valid virtual environments presented via a head-mounted display. We examined in 27 low (LSA) and 26 high socially anxious (HSA) individuals fear ratings, physical behavior (duration of approach), hypervigilance (time to first fixation), and attentional avoidance (count of fixations) toward virtual female and male agents (CS) during social fear conditioning (SFC) and extinction in virtual reality (VR). As hypothesized, generally SFC was successfully induced and extinguished concerning the fear ratings. Our findings partly support the vigilance-avoidance hypothesis as HSA directed especially at the first half of the fear acquisition their initial attention more at CS+ than CS− agents, and avoided subsequently the CS+ more than the CS− agents during the fear acquisition. In contrast, in LSA participants initial and sustained attention did not differ between CS+ and CS− agents during fear acquisition. We conclude that HSA individuals guide their initial attention to emotionally threatening stimuli and subsequently avoid the threatening stimuli to possibly reduce their emotional distress, whereas LSA individuals regulate themselves less in their (fear) responses during SFC. Measuring implicit gaze behavior within a well-controlled virtual environment is an interesting innovative tool to in deeply investigate the impact of attention on emotional learning processes.

## Introduction

A lot of research has investigated selective attention that describes social anxiety to conceive the architecture of information processing in social anxiety disorder (SAD). The most popular theoretical models of SAD are the cognitive model from Clark and Wells (1995) and the cognitive-behavioral model from Rapee and Heimberg (1997). Both models comprise biases in information processing that develop and/or maintain SAD and implicate the importance of selective attention (e.g., vigilance, avoidance) in social anxiety (Schofield et al., 2012).

Clark and Wells (1995) suggest that central factors of information processing in SAD include increased self-focused attention, safety behavior, and problematic anticipatory and subsequent processing. For example, avoiding attention toward feared stimuli during social interactions serves as safety behavior to regulate the internal distress (e.g., reducing eye contact), to avert feared negative situations (e.g., assessments), and/or to avoid real or perceived feared social appraisal of others as an attempt of self-regulation. Accordingly, conscious attentional avoidance of salient feared stimuli is active during social evaluations and is assumed to maintain SAD.

In comparison, Rapee and Heimberg (1997) focus more on vigilance as selective attention toward emotionally negative information in SAD. The authors emphasize that socially anxious persons utilize an exaggerated attentional allocation toward any sign of impending negative feedback of others, which can lead to biased estimations of a more threatening social environment and that a wisp of negative evaluation will be perceived rapidly and reinforce the threatening negative self-evaluation (Heimberg et al., 2014).

Chen and Clarke (2017) report in their review that there are different results in empirical research among socially anxious humans with attentional bias to relevant emotionally threat stimuli (Gilboa-Schechtman et al., 1999; Perowne and Mansell, 2002; Lange et al., 2011; Çek et al., 2016; Lazarov et al., 2016) and who also exhibited attentional avoidance toward socially feared information (Chen et al., 2002; Wieser et al., 2009b; Singh et al., 2015; Shechner et al., 2017). Mogg and Bradley (2002) suggest that vigilant patterns of attention may be located in initially attentional processing. Furthermore, Chen and Clarke (2017) conclude that there is an association between social anxiety and vigilance (e.g., hyperscanning the environment) as well as attentional avoidance (e.g., reducing eye contact) according to the information processing of emotionally social stimuli.

These mixed empirical results caused the formulation of a vigilance-avoidance hypothesis of selective attention (Bögels and Mansell, 2004; Wieser et al., 2009a, b; Chen and Clarke, 2017). It assumes that socially anxious humans guide their initial attention to emotionally threatening information (hypervigilance) and avoid the negative information subsequently (attentional avoidance) to reduce emotional distress (Bögels and Mansell, 2004; e Claudino et al., 2019).

In recent years the rising use of eye-tracking technology offered novel insights into diverse aspects of selective attention that are responsible in the etiology and maintenance of SAD (Lange et al., 2011; Schofield et al., 2012; Lazarov et al., 2016; Chen and Clarke, 2017). Furthermore, patterns of gaze behavior could be used as sensitive index of fear learning (Hopkins et al., 2015). Using an eye-tracking device allows us to directly and continuously measure visual selective attention in real time. This methodology registers the precise location of eye movement and gaze behavior over the course of time as a relatively naturalistic assessment of attention without an explicit response of the participant. Bögels and Mansell (2004) described in their review a lot of paradigms like the emotional Stroop task (Maidenberg et al., 1996; Becker et al., 2001), visual search task (Rinck et al., 2003), modified dot-probe task (Mansell et al., 2002; Mogg et al., 2004), and eye-tracking tasks (Garner et al., 2006) to investigate hypervigilance and/or avoidance in the information processing of selective attention in social anxiety.

Relating to the nature of SAD, one major deficiency of these experimental paradigms is that participants observe stimuli with a low ecological validity (e.g., images of faces or words on a screen). Consequently, more research is necessary to validate how socially anxious persons respond to more ecological valid socially relevant stimuli. Empirical studies on gaze behavior in social interactions (e.g., interviews or having a speech causing fear of negative evaluation) are rare and have methodical shortcomings, e.g., less valid and reliable measurement of dependent variables and reduced control of independent variables in real interactions (Mühlberger et al., 2008; Wieser et al., 2009a).

Several empirical studies with an innovative technology such as virtual reality (VR) investigated social interaction processes with good experimental control, high ecological validity, low time costs, and highly valid assessments of dependent variables like physical and gaze behavior. Further advantages are the systematic and independent manipulations of eye-gaze directions or gender of the interaction partner as well as the presented social situations, all of which are either very costly or difficult to control *in vivo*. Most importantly, eye and head movements or interpersonal space between the participant and the counterpart can simply be recorded with the use of eye- and head-tracking within VR (Mühlberger et al., 2008; Wieser et al., 2009a, b; Dechant et al., 2017; Reichenberger et al., 2019). This innovative technology gives us the opportunity to assess directly approach-avoidance behavior in social interactions as well.

Wieser et al. (2010) investigated the impact of sex, gaze, and interpersonal distance (0.5 m vs. 1.5 m) on social anxiety in women in a virtual social interaction scenario. Socially anxious women showed attentional avoidance in response to virtual male counterparts, which were farther away and showed a straight gaze. In addition, they revealed more backward movements of the head (avoidance behavior) toward male agents, regardless of the interpersonal space. Moreover, Dechant et al. (2017) reported that HSA presented more attentional avoidance than LSA participants in a virtual social interaction paradigm. As we know, avoidance behavior is a key element in the maintenance of anxiety and also in fear learning.

In our previous study, we investigated differences between the gender and the influence of female vs. male agents in *N* = 60 high and low socially anxious individuals concerning social fear conditioning (SFC) and extinction in VR (Reichenberger et al., 2019). This SFC paradigm used social interactions in a standardized and experimentally controlled way, and disorder-relevant US (spitting simulated by an aversive air blast and verbal rejection) to examine affective learning in social anxiety. We measured with an enhanced ecological validity the experience, psychophysiology, behavior and cognition in emotional learning processes corresponding on each level of emotional reactions. Besides the successfully induced and extinguished SFC, we could present higher social fear conditionability in women, but participants reported no higher fear while approached male than female agents. Interestingly, we found enhanced fear responses in the fear-potentiated startle to male than to female agents which might indicate higher social fear conditionability toward male persons. In addition, high socially anxious women revealed more behavioral avoidance to male than to female agents during fear acquisition. In comparison, HSA men did not discriminate between male or female agents. We concluded that the gender of the participants rather affect reflective processes (e.g., reported fear as well as contingency and skin conductance response), while the gender of the virtual agents influence more automatic measures (e.g., fear-potentiated startle and behavioral avoidance). Besides these self-reported and psychophysiological measures, binocular gaze behavior was also continuously recorded.

In the current study, we analyzed the recorded physical and gaze behavior (hypervigilance and attentional avoidance) during the SFC paradigm in a subsample from Reichenberger et al. (2019). Therefore, we aim to investigate the effect of induced and extinguished social fear on physical behavior (duration of approach) as well as hypervigilance (time of the first fixation) and attentional avoidance (count of fixations) toward female and male agents in HSA and LSA students in the SFC paradigm in VR. Based on the aforementioned empirical results, we hypothesize that (1) hypervigilance and attentional avoidance for CS+ would increase compared to CS− during fear acquisition. (2) In addition, we hypothesize that HSA will show enhanced hypervigilance to CS+ compared to CS− and will avoid more the CS+ compared to CS− than LSA participants during fear acquisition. At least, we expected that hypervigilance and attentional avoidance for CS+ would return to baseline levels and won’t differ to CS− during fear extinction.

## Materials and Methods

### Participants

One hundred and eighty Psychology and Media Informatics Science students at the University of Regensburg filled in a pre-screening questionnaire consisting of demographic and exclusion criteria as well as social anxiety using the German version of the Social Phobia Inventory (SPIN; Stangier and Steffens, 2002). Exclusion criteria were age <18 or >55 years, an existing or former diagnosed neurological or mental disorder (excepting SAD), a current neurological, psychiatric or psychotherapeutic treatment, a history of psychotropic drug use, pregnancy or lactation, and an attendance in a previous SFC study (Reichenberger et al., 2019).

Based on our previous study investigating SFC and extinction in VR (Reichenberger et al., 2017) a sample size of 54 was estimated with G^∗^Power 3.1.9.2 to detect medium effects (*f* = 0.25) at power = 0.95 and α = 0.05. The current study consists of a subsample from Reichenberger et al. (2019). However, on the basis of eye-tracking failure during data acquisition, we had to exclude seven participants from the original sample of *n* = 60. Thus, the current study contained a sample of 53 undergraduate students (27 LSA: 51.85% female, aged between 18 and 43; and 26 HSA: 53.85% female, aged between 18 and 33). The SPIN cut-off value of 19 differentiates patients with SAD and healthy persons with a diagnostic accuracy of 79% (Connor et al., 2000). A successful segmentation into a HSA and LSA group was given, since HSA reveal significantly higher scores than LSA participants with quite large between group effect sizes in the SPIN and in the German version of the Social Interaction Anxiety Scale (SIAS; Stangier et al., 1999). Please see Table 1 for the questionnaire data. In addition, the groups did not differ in age [*t*(51) = 0.832, *p* = 0.409] and sex ratio [HSA: 12 men; LSA: 13 men; χ^2^(1, 53) = 0.021, *p* = 0.884]. The final sample was free of any neurological or mental disorder (self-report) and had unimpaired or corrected hearing and vision. All of the students received course credit as reimbursement for their attendance. The Ethics Committee of the University of Regensburg approved the study.

**TABLE 1:** Questionnaire data.

	LSA (*n* = 27)		HSA (*n* = 26)				
					
	*M*	*SD*	*M*	*SD*	*t*	*p*	*d*
SPIN	10.8	3.82	27.3	7.06	–10.624	<0.001	2.98
SIAS	16.2	10.1	29.5	11.1	–4.585	<0.001	1.28
PRF-D	9.78	3.97	5.31	3.74	4.219	<0.001	1.18
SBS	22.6	8.18	27.9	6.51	–2.636	0.011	0.74
VIG	10.7	3.63	13.5	2.69	–3.007	0.004	0.84
CAV	10.4	3.63	9.15	2.63	1.392	0.170	0.25

*Means (*M*) and standard deviations (*SD*) and also *t*- and *p*-values and Cohen’s *d* as well as number of participants (*n*) are given for low (LSA) and high socially anxious (HSA) participants for questionnaire data. SPIN = Social Phobia Inventory; SIAS = Social Interaction Anxiety Scale; PRF-D = German Personality Research Form – Dominance Scale; SBS = Submissive Behavior Scale; VIG = Vigilance of the Mainz Coping Inventory; CAV = Cognitive Avoidance of the Mainz Coping Inventory.*

### Apparatus

The VR was displayed with the HTC VIVE head-mounted display (HMD; HTC Corporation, Taoyuan, Taiwan) and was generated via the Steam Source engine (Valve Corporation, Bellevue, WA, United States). The presented virtual environment was controlled by CyberSession Research 5.6 (VTplus GmbH, Würzburg, Germany). Eye-tracking was continuously recorded binocular with a sampling rate of 250 Hz by the SMI (SensoMotoric Instruments, Boston, United States) head gear integration into the HMD. The trackable field of view is 110° with an accuracy of typ. 0.2° for eye-gaze vectors. CyberSession Research submits the processing of eye-tracking data in log files. All of the sounds were presented over headphones (Sennheiser HD-215, Sennheiser electronic GmbH, Germany). The participants could move in the virtual environment using a joystick (Logitech Extreme 3D Pro Joystick, Logitech GmbH, Germany).

**FIGURE 1:**
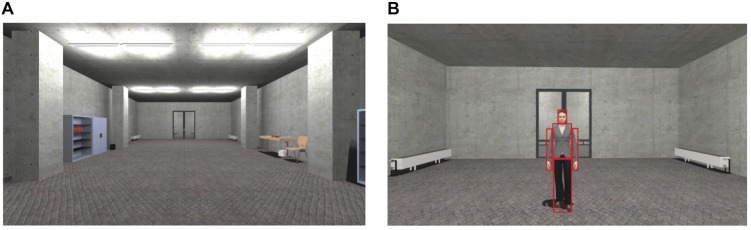
Virtual environment and stimuli. **(A)** Starting point in the room in which the learning phases took place. **(B)** Social stimuli (agents) used for social fear conditioning (SFC) with the dynamic social area of interest (red rectangles). For interpretation of the references to color in this figure, the reader is referred to the web version of this article.

During the study participants were immersed into a virtual room, which was modeled after a corridor of the University of Regensburg (see Figure 1A). In all three phases (pre-acquisition, acquisition, and extinction), the starting position was at one end of the room and at the opposite end a female or male agent was presented. The agents moved their head and upper body slightly and gazed dynamically at the participant to appear alive (please see Reichenberger et al., 2019). The task of the participants was to actively approach in all three phases the female and male agents until a distance of 30 cm where movement stopped. In 75% of the SFC trials female and male agents (CS+) were paired with an aversive unconditioned stimulus (US) existing of a sound of spitting attended by an air blast toward the right neck of the participant (5 bar, 10 ms) followed by the verbal rejection “Get lost!.” The agents’ facial expression was adapted to the spitting and verbal rejection. The other female and male agent (CS−) was not paired with an US. The pre-acquisition and extinction phase proceeded in exactly the same way as the fear conditioning, except for presenting no US and the appearance of a neutral agent (NS) of both genders in the extinction phase. The order of the agents was pseudo-randomized in each phase (see Shiban et al., 2015; Reichenberger et al., 2017, 2019).

### Measures

At the beginning of the experiment, participants completed the following questionnaires: a socio-demographic questionnaire, the SPIN, the SIAS, the Dominance scale of the German Personality Research Form (PRF-D; Stumpf et al., 1985), the Submissive Behavior Scale (SBS; Allan and Gilbert, 1997), and the subtest Ego threat of the German version of the Mainz Coping Inventory (MCI; Krohne and Egloff, 1999) with the two coping strategies Vigilance (VIG) and Cognitive Avoidance (CAV). A detailed description of the questionnaires is given in our previous study (see Reichenberger et al., 2019).

During the VR, we quantified the experienced anxiety in regard of each agent with verbally ratings (“On a scale from 0 to 100, how intense is your anxiety during the presence of this person?”) in the rating phases which followed each of the three phases. Participants approached each of the diverse agents until they reached the specific distance of 30 cm to the agents, lights faded out and they verbally rate their anxiety.

Besides these self-reported measures, physical behavior (duration to approach) and binocular gaze behavior was continuously recorded during the SFC paradigm. To ensure that the gaze behavior was measured correctly by the SMI integration eye-tracking device, a 5-point calibration was conducted before the learning phases in VR. The continuous gaze behavior was analyzed as the time to first fixation (hypervigilance) and the mean percentage of the fixation counts (attentional avoidance) in predefined dynamic social areas of interest (AOI) for each agent (CS+, CS−, and NS) in each phase (pre-acquisition, acquisition, extinction). The social AOI was defined by a rectangle around the face and the body of each agent separately (see Figure 1B).

### Procedure

**FIGURE 2:**
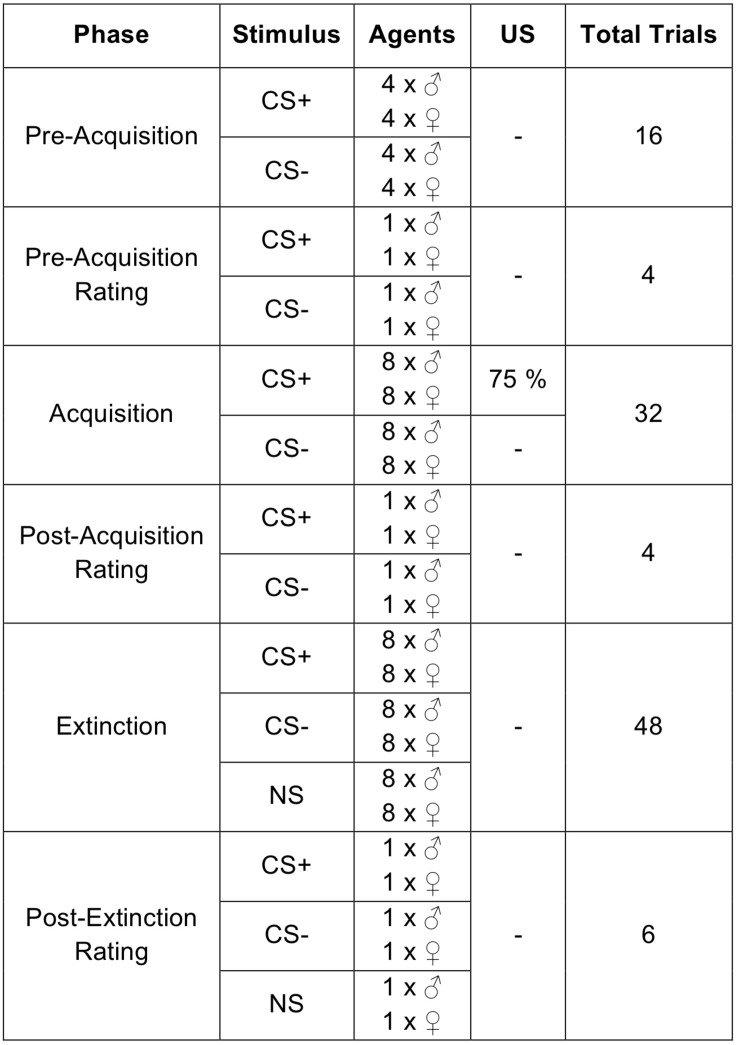
Experimental procedure. As unconditioned stimulus (US) a sound of spitting attended by an air blast followed by the verbal rejection was applied. The order of the agents was pseudorandomized in each phase. CS+ = agent paired with aversive US; CS− = agent without aversive US; NS = agent without aversive US and only appearing during the extinction phase.

The study consisted of briefing the participants in written form, signing the informed consent, filling out questionnaires, and the SFC paradigm in VR (see Figure 2). At the beginning of the VR, participants could explore the virtual environment and learn how to navigate with the joystick. Afterward, the recorded instruction “You will now meet several human beings. Please try to move directly toward the persons until they are right in front of you” was presented. Therefore, participants approached female and male agents actively until the specific distance of 30 cm to the agents, lights faded out, the participants were returned to the starting point and the next trial with a new agent at the opposite end of the room was presented. Which agent was presented as CS+ or CS− was balanced across participants.

In the pre-acquisition phase each female and male CS+/CS− agent was presented four times, resulting in 16 total trials and the US was not presented yet. Following this, the first rating phase took place for each female and male CS+/CS− agent.

The acquisition phase consisted of 32 trials in total. Each female and male CS+/CS− agent was presented eight times. Only the conditioned agent (CS+) was paired with 75% contingency of the US during the acquisition phase.

Following the fear conditioning, the second rating phase took place for each agent. After that, participants had a 5 min break where they took off the HMD and had the possibility to sit down and close their eyes.

After the break, the extinction phase took place exactly the same way as the fear acquisition, except for the absence of the US as well as the presentation of one additional female and one additional male NS, resulting in 48 total trials. At least, the third rating phase took place for each female and male CS+, CS−, and NS agent. A more detailed description of the procedure is given by Reichenberger et al. (2019).

### Data Reduction and Statistical Analyses

We utilized MATLAB 9.5 (MathWorks Inc., Germany) to analyze the behavioral data and SPSS 25.0 (IBM Corp., Armonk, NY, United States) to perform further analyses of the subjective and behavioral variables.

The mean fear rating for each female and male agent (CS+, CS−, NS) measured after each phase (pre-acquisition, acquisition, and extinction) were calculated. Investigating changes in fear ratings concerning SFC, two repeated-measures ANOVAs with the within-subject factors time (pre vs. post-acquisition for acquisition, and post-acquisition vs. post-extinction for extinction), stimulus (CS+ vs. CS−) and agent (female vs. male), and the between-subject factors gender (women vs. men) and social anxiety (LSA vs. HSA) were computed.

For the statistical analyses of the behavioral outcome variables, we defined that one trial consisted of the onset (as soon as the participant starts to approach using the joystick) and the offset (30 cm distance to the agent) in each phase.

For the physical behavior (duration of approach) as well as both gaze behavioral outcome variables, we computed means for the pre-acquisition phase, while the first four and the last four approaches in the acquisition and the extinction phase were calculated as the means of the beginning and the end of the acquisition as well as extinction phase, respectively.

Hypervigilance was measured by the time to first fixation on a social AOI in a trial. Since the duration of approach toward the agents was different between the phases and both groups (see Figure 4), attentional avoidance was calculated by the mean percentage of the count of fixations (number of fixations in the social AOI divided by the total number of fixations in the trial). The social AOI contain the face and the body of each agent in each approach trial. Fixations were defined based on a spatial (a diameter of 1° visual angle) and a temporal criterion (a minimum of 150 ms). Gaze behavior data were excluded if for more than 20% of the sampling points of a trial the eye-tracker couldn’t identify the gaze direction of the participant.

Checking for potential distinctions in pre-acquisition, ANOVAs with the within-subject factors stimulus (CS+ vs. CS−) and agent (female vs. male) and the between-subject factors gender (women vs. men) and social anxiety (low vs. high) were calculated for each behavioral outcome variable. In order to analyze conditioning effects, repeated-measures ANOVAs with the within-subject factors time (beginning vs. end), stimulus (CS+ vs. CS−) and agent (female vs. male) and the between-subject factors gender (women vs. men) and social anxiety (LSA vs. HSA) were calculated for the acquisition and extinction phase. Testing for possible generalization effects, ANOVAs with the within-subject factors stimulus (CS+ vs. CS− vs. NS) and agent (female vs. male) and the between-subject factors gender (women vs. men) and social anxiety (low vs. high) were calculated at the beginning of the extinction phase as well.

All of the significant interactions were performed by separate follow-up ANOVAs or Student’s *t*-tests. Partial η^2^ ($\eta_{p}^{2}$) scores and Cohen’s *d* served as indices of effect size. All statistical analyses utilized a *p* < 0.05 as level of statistical significance.

## Results

As expected, significant group differences (see Table 1) were found in social anxiety (SPIN), in anxiety of social interactional situations (SIAS), in dominant (PRF-D) and submissive behavior (SBS), and vigilance (VIG). However, groups did not differ in the total score of cognitive avoidance (CAV). As this is a subsample of the study published by Reichenberger et al. (2019), results in these analyses reflect the earlier results based on the whole sample.

### Self-Report

**FIGURE 3:**
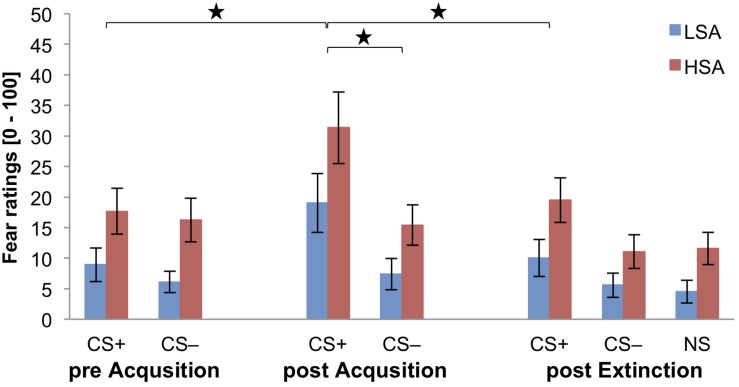
Fear ratings (*n* = 53) for CS+, CS−, and NS in the three rating phases for low (LSA) and high socially anxious (HSA) participants. CS+ = agent paired with aversive unconditioned stimulus (US); CS*–* = agent without aversive US; NS = agent without aversive US and appearing only in the extinction phase; pre Acquisition = before acquisition phase; post Acquisition = after acquisition phase; post Extinction = after extinction phase. Mean fear ratings (0 = very low fear to 100 = very high fear) were given. Significant differences are indicated with an asterisk. Standard errors are presented by error bars.

Figure 3 displays that in each phase all fear ratings are higher for HSA than for LSA participants. After pre-acquisition, both stimuli are rated almost equal in both groups. After fear acquisition, the self-reported fear for CS+ agents is clearly higher than for CS− agents in both groups. After fear extinction, the ratings for CS+ agents decrease and the self-reported fear for CS− and NS agents do not distinguish after fear extinction, whereas the CS+ agents are rated slightly higher in both groups.

For fear acquisition, an ANOVA comparing the self-reported fear pre and post acquisition revealed a significant effect of Time × Stimulus × Gender × Social Anxiety (please see Table 2 for all significant results of the ANOVA). Follow-up ANOVAs were performed for the HSA and LSA group separately to disentangle this fourfold interaction. For the HSA group, a significant effect of Time × Stimulus × Gender could be approved. Follow-up ANOVAs were conducted for HSA women and men separately. For HSA women, a significant interaction effect of Time × Stimulus could be found and the follow-up *t*-tests indicate that the self-reported fear increased significantly for CS+, *t*(13) = −3.35, *p* = 0.005, *d* = 0.69, but not for CS− (*p* = 0.313). In comparison, for HSA men a significant main effect of Time *F*(1,11) = 4.83, *p* = 0.050, $\eta_{p}^{2}$ = 0.31, Stimulus *F*(1,11) = 4.88, *p* = 0.049, $\eta_{p}^{2}$ = 0.31, but not for Time × Stimulus *F*(1,11) = 2.83, *p* = 0.121, $\eta_{p}^{2}$ = 0.20, was detected. Exploratory *t*-tests might give a first hint that also in men the self-reported fear might increase for CS+, *t*(11) = −2.29, *p* = 0.043, *d* = 0.5, but not for CS− (*p* = 0.237). For the LSA group, we found significant interaction effects for Time x Stimulus, and the follow-up *t*-tests show that the subjective fear increased significantly for CS+, *t*(26) = −3.91, *p* < 0.001, *d* = 0.51, but not for CS− (*p* = 0.424) as expected. In sum, the findings of the self-reported fear reveal that successful SFC took place like in the whole sample by Reichenberger et al. (2019), with the exception of HSA men.

**TABLE 2:** Significant results of the ANOVA for fear ratings of the acquisition and extinction phase.

Effect	*df*	*F*	$\eta_{p}^{2}$	*p*
**Acquisition**				
*Total*				
Time	1, 49	18.9	0.28	<0.001
Stimulus	1, 49	23.5	0.32	<0.001
Gender	1, 49	5.13	0.10	0.028
Social Anxiety	1, 49	4.30	0.08	0.043
Time × Stimulus	1, 49	25.8	0.35	<0.001
Time × Stimulus × Gender × Social Anxiety	1, 49	4.13	0.08	0.048
*LSA*				
Time	1, 25	11.8	0.32	0.002
Stimulus	1, 25	12.0	0.32	0.002
Time × Stimulus	1, 25	10.7	0.30	0.003
*HSA*				
Time	1, 24	8.05	0.25	0.009
Stimulus	1, 24	11.6	0.33	0.002
Gender	1, 24	4.29	0.15	0.049
Time × Stimulus	1, 24	14.9	0.38	<0.001
Time × Stimulus × Gender	1, 24	5.62	0.19	0.026
*HSA women*				
Stimulus	1, 13	8.64	0.40	0.011
Time × Stimulus	1, 13	13.7	0.51	0.003
*HSA men*				
Time	1, 11	4.83	0.31	0.050
Stimulus	1, 11	4.88	0.31	0.049
**Extinction**				
*Total*				
Time	1, 49	28.0	0.36	<0.001
Stimulus	1, 49	28.6	0.37	<0.001
Agent	1, 49	4.77	0.09	0.034
Gender	1, 49	5.12	0.10	0.028
Stimulus × Gender	1, 49	4.88	0.09	0.032
Time × Stimulus	1, 49	16.6	0.25	<0.001
Time × Stimulus × Agent	1, 49	5.89	0.11	0.019
*Female agents*				
Time	1, 49	24.8	0.34	<0.001
Stimulus	1, 49	20.9	0.30	<0.001
Time × Stimulus	1, 49	23.3	0.32	<0.001
*Male agents*				
Time	1, 49	25.9	0.35	<0.001
Stimulus	1, 49	21.1	0.30	<0.001
Time × Stimulus	1, 49	5.73	0.11	0.021

**df* = degrees of freedom; $\eta_{p}^{2}$ = effect size; Time = pre vs. post acquisition for acquisition and post acquisition vs. post extinction for extinction; Stimulus = CS+ vs. CS−; Agent = female vs. male agent; Gender = women vs. men; Social Anxiety = LSA vs. HSA.*

Concerning fear extinction, an ANOVA comparing fear ratings pre and post extinction approved significant effects of Stimulus × Gender, and Time × Stimulus × Agent (please see Table 2). Follow-up ANOVAs were performed for female and male agents separately to unravel this threefold interaction. For female agents, a significant interaction effect of Time × Stimulus could be found and follow-up *t*-tests detect that the self-reported fear significantly decreased for the CS+, *t*(52) = 5.57, *p* < 0.001, *d* = 0.53, but not clearly for the CS−, *t*(52) = 1.99, *p* = 0.051, *d* = 0.12. For male agents, a significant interaction effect of Time × Stimulus was identified and follow-up *t*-tests highlight that the fear ratings of the CS+, *t*(52) = 4.61, *p* < 0.001, *d* = 0.36, and CS−, *t*(52) = 3.23, *p* = 0.002, *d* = 0.31, significantly decreased. For the Stimulus × Gender interaction, follow-up *t*-tests reveal that women rated the CS+ significantly higher than men, *t*(45.7) = 2.48, *p* = 0.017, *d* = 0.68, however, there was no difference for the CS− (*p* = 0.096). The results of the fear ratings indicate that social fear extinction was also successful in the subsample as it was in the sample reported by Reichenberger et al. (2019). However, no generalization effect was found.

### Physical Behavior

**FIGURE 4:**
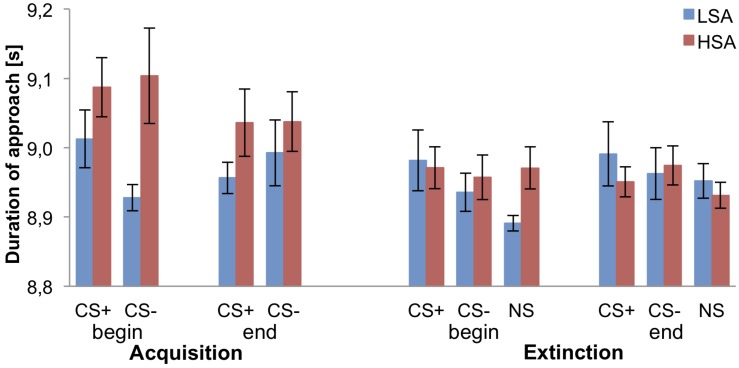
Mean duration of approach for low (LSA) and high socially anxious (HSA) participants (*n* = 53). CS+ = agent paired with aversive unconditioned stimulus (US); CS− = agent without aversive US; NS = agent without aversive US and only appearing during the extinction phase. Standard errors are presented by error bars.

Figure 4 displays that at the beginning of the fear acquisition the LSA approach the CS− faster than the CS+, whereas the HSA don’t differ between both agents. Interestingly, during fear acquisition HSA need slightly more time to approach the CS+ and CS− agents than the LSA group. During fear extinction, the duration of approach toward the agents differs not between both groups, besides the duration of approach toward the NS agent was shorter for the LSA than the HSA group at the beginning of the extinction.

Investigating differences in the pre-acquisition phase an ANOVA confirmed only a significant effect of Agent, *F*(1,49) = 5.48, *p* = 0.023, $\eta_{p}^{2}$ = 0.10, indicating a longer approach time toward male (*M* = 9.08, *SD* = 0.25) than female (*M* = 9.01, *SD* = 0.20) agents.

Relating to the fear acquisition, we detected no significant effect for Social Anxiety, *F*(1,48) = 3.08, *p* = 0.086, $\eta_{p}^{2}$ = 0.06, and Time × Stimulus × Social Anxiety, *F*(1,48) = 3.02, *p* = 0.088, $\eta_{p}^{2}$ = 0.06, but a significant effect for Agent × Gender × Social Anxiety, *F*(1,48) = 4.38, *p* = 0.042, $\eta_{p}^{2}$ = 0.08. Follow-up ANOVAs were conducted for both HSA and LSA participants separately, but no main or interaction effect reached significance. For LSA, the effect of Agent × Gender, *F*(1,24) = 3.57, *p* = 0.071, $\eta_{p}^{2}$ = 0.13, and Time × Stimulus, *F*(1,24) = 4.13, *p* = 0.053, $\eta_{p}^{2}$ = 0.15, narrowly missed significance. With regard to fear conditioning, exploratory *t*-tests might give a preliminary hint that at the first part of the fear acquisition the approaching time toward the CS+ might be higher than to the CS−, *t*(25) = 2.091, *p* = 0.047, *d* = 0.53, whereas at the end of the fear acquisition the difference of the approaching time seems vanished (*p* = 0.415).

For fear extinction, a significant main effect of Time × Stimulus × Agent × Gender, *F*(1,49) = 6.52, *p* = 0.014, $\eta_{p}^{2}$ = 0.12, was found. Follow-up ANOVAs were performed for both genders separately, but no significant main or interaction effect was found. Furthermore, no generalization effect was found.

### Hypervigilance

**FIGURE 5:**
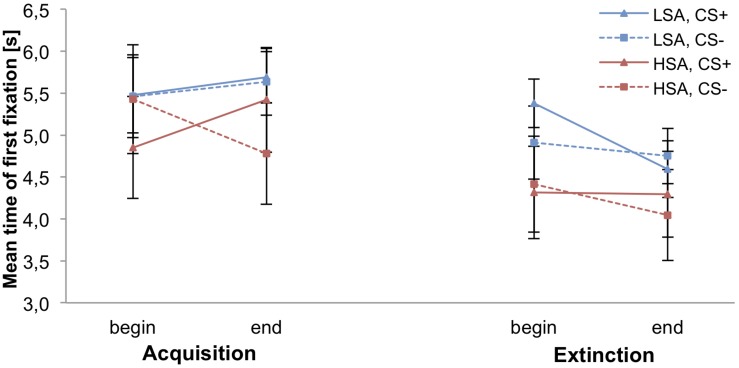
Mean time of the first fixation within the social area of interest (agent) for low (LSA) and high socially anxious (HSA) participants (*n* = 53). CS+ = agent paired with aversive unconditioned stimulus (US); CS− = agent without aversive US. Standard errors are presented by error bars.

Figure 5 shows that at the beginning of the fear acquisition HSA fixate more early the CS+ than the CS− agent. In comparison, at the end of the fear acquisition HSA show a lower time of the first fixation to the CS− compared to the CS+ agent. The LSA show a small increase of the time of the first fixation toward both agents during fear acquisition. In the extinction phase, we can detect a decrease of the time of the first fixation toward the agents in both groups.

Proving differences before fear conditioning, an ANOVA confirmed no significant differences during pre-acquisition. For fear acquisition, an ANOVA revealed a significant effect of Time × Gender, *F*(1,46) = 4.59, *p* = 0.037, $\eta_{p}^{2}$ = 0.10, Time × Stimulus, *F*(1,46) = 6.06, *p* = 0.018, $\eta_{p}^{2}$ = 0.12, and Time × Stimulus × Social Anxiety, *F*(1,46) = 4.06, *p* = 0.050, $\eta_{p}^{2}$ = 0.08. Follow-up ANOVAs were conducted for the HSA and LSA group separately. For HSA, the interaction effect of Time × Stimulus, *F*(1,24) = 12.73, *p* = 0.002, $\eta_{p}^{2}$ = 0.35, reached significance. Follow-up *t*-tests indicate that at the beginning (first half) of the acquisition phase the time till the first fixation toward the CS+ was significantly lower than to the CS−, *t*(25) = −2.377, *p* = 0.025, *d* = 0.18, whereas at the end (second half) of the acquisition phase the time till the first fixation toward the CS+ was significantly higher than to the CS−, *t*(25) = 2.687, *p* = 0.013, *d* = 0.21. For LSA, no significant main or interaction effects were found.

Regarding fear extinction, an ANOVA detected significant effects of Time, *F*(1,48) = 7.01, *p* = 0.011, $\eta_{p}^{2}$ = 0.13, and Stimulus × Agent, *F*(1,48) = 10.75, *p* = 0.002, $\eta_{p}^{2}$ = 0.18. Follow-up *t*-tests reveal a significant lower time till the first fixation toward the male CS− compared to the female CS−, *t*(52) = −3.641, *p* < 0.001, *d* = 0.29, but no significant difference between the male and female CS+ (*p* = 0.125). However, no generalization effect was found.

### Attentional Avoidance

**FIGURE 6:**
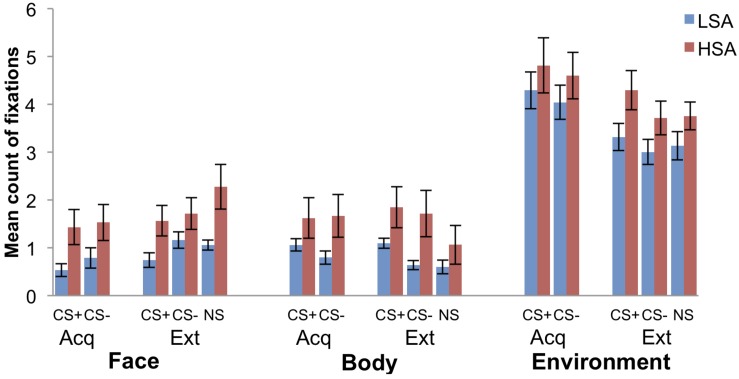
Mean count of fixations within the social area of interest (face and body) as well as environment for low (LSA) and high socially anxious (HSA) participants (*n* = 53). CS+ = agent paired with aversive unconditioned stimulus (US); CS− = agent without aversive US; NS = agent without aversive US and appearing only in the extinction phase; Acq = acquisition phase; Ext = extinction phase. Standard errors are presented by error bars.

**FIGURE 7:**
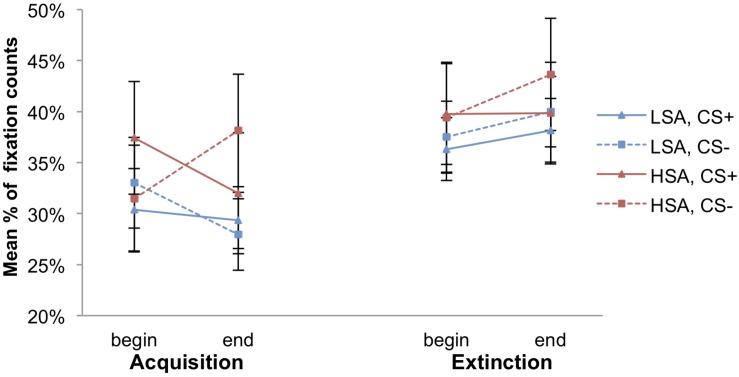
Mean percentage of the count of fixations within the social area of interest (agent) for low (LSA) and high socially anxious (HSA) participants (*n* = 53). CS+ = agent paired with aversive unconditioned stimulus (US); CS− = agent without aversive US. Standard errors are presented by error bars.

As we can see, the mean count of fixations toward the face and the body of the agents (social AOI), as well as toward the environment is higher for the HSA compared to the LSA participants (see Figure 6). Figure 7 displays the mean percentage of the count of fixations that contain the given social AOI (agent) for LSA and HSA participants during fear acquisition and extinction. In the fear acquisition phase, HSA show more attentional avoidance to CS+ than to CS−, whereas LSA avoid both CS+ and CS− in the same level. In the extinction phase, we can detect a higher increase of fixations to CS− than to CS+.

Checking differences in the pre-acquisition phase, an ANOVA showed a significant effect of Stimulus × Agent × Gender, *F*(1,46) = 7.33, *p* = 0.009, $\eta_{p}^{2}$ = 0.14, but follow-up ANOVAs conducting for women and men separately detected no significant main or interaction effects for both gender.

Concerning fear acquisition, an ANOVA approved significant effects of Time × Gender, *F*(1,46) = 4.76, *p* = 0.034, $\eta_{p}^{2}$ = 0.09, Time × Stimulus × Social Anxiety, *F*(1,46) = 8.51, *p* = 0.005, $\eta_{p}^{2}$ = 0.16, and Stimulus × Agent × Social Anxiety, *F*(1,46) = 6.20, *p* = 0.016, $\eta_{p}^{2}$ = 0.12. Follow-up ANOVAs were conducted for the HSA and LSA group. For HSA, the interaction effects of Stimulus × Agent, *F*(1,24) = 9.84, *p* = 0.004, $\eta_{p}^{2}$ = 0.29, and Time × Stimulus, *F*(1,24) = 12.5, *p* = 0.002, $\eta_{p}^{2}$ = 0.34, reached significance. For the Stimulus × Agent interaction, follow-up *t*-tests show that HSA fixated significantly more frequently the female than the male CS+, *t*(25) = −2.51, *p* = 0.019, *d* = 0.22, whereas between the female and the male CS− was no significant difference (*p* = 0.170). Regarding the Time × Stimulus interaction, follow-up *t*-tests reveal that HSA looked significantly more frequently toward the CS+ than to the CS− at the beginning of the fear acquisition, *t*(25) = 2.39, *p* = 0.025, *d* = 0.22, whereas at the end of the acquisition they exhibited significantly more fixations counts toward the CS− than to the CS+, *t*(25) = −2.98, *p* = 0.006, *d* = 0.22, indicating an effect of fear conditioning according the attentional avoidance in HSA. For LSA, we detected no significant main or interaction effect.

For fear extinction, an ANOVA showed significant effects of Stimulus x Agent, *F*(1,48) = 16.5, *p* < 0.001, $\eta_{p}^{2}$ = 0.26, Time × Agent × Social Anxiety, *F*(1,48) = 4.63, *p* = 0.037, $\eta_{p}^{2}$ = 0.09, and Stimulus × Agent × Gender × Social Anxiety, *F*(1,48) = 4.96, *p* = 0.031, $\eta_{p}^{2}$ = 0.09. To disentangle this threefold interaction, we conducted follow-up ANOVAs for HSA and LSA separately. For HSA, significant effects of Stimulus × Agent, *F*(1,24) = 21.5, *p* < 0.001, $\eta_{p}^{2}$ = 0.47, and Stimulus × Agent × Gender, *F*(1,24) = 9.17, *p* = 0.006, $\eta_{p}^{2}$ = 0.28, were identified. Another follow-up ANOVAs were performed for HSA women and men separately. For HSA women, no significant main or interaction effect was detected. For HSA men, we found a significant interaction effect of Stimulus × Agent, *F*(1,11) = 17.1, *p* = 0.002, $\eta_{p}^{2}$ = 0.61, and the follow-up *t*-tests show that HSA men fixated significantly more female than male CS+ agents, *t*(11) = −3.17, *p* = 0.009, *d* = 0.9, whereas no significant difference between the female and male CS− was identified (*p* = 0.059). With regard to LSA, we found significant effects of Gender, *F*(1,24) = 8.21, *p* = 0.009, $\eta_{p}^{2}$ = 0.26, Time × Agent, *F*(1,24) = 6.76, *p* = 0.016, $\eta_{p}^{2}$ = 0.22, and Time × Stimulus × Agent × Gender, *F*(1,24) = 4.77, *p* = 0.039, $\eta_{p}^{2}$ = 0.17. Therefore, another follow-up ANOVAs were conducted for LSA women and men separately. No significant main or interaction effect was found for LSA women. For LSA men, a significant interaction effect of Time × Agent *F*(1,12) = 6.94, *p* = 0.022, $\eta_{p}^{2}$ = 0.37, could be detected and follow-up *t*-tests reveal that the fixations toward female agents significantly increased, *t*(12) = −2.24, *p* = 0.045, *d* = 0.94, whereas the fixations toward the male agents did not change during fear extinction (*p* = 0.814). However, no generalization effect was found.

## Discussion

The present study is one of the first to investigate social anxiety related physical behavior as well as hypervigilance and attentional avoidance through the use of implicit eye-tracking in HSA and LSA participants (1) within the context of SFC, (2) with the use of anthropomorphic stimuli (3) in a social interaction with enhanced ecological validity (4) in VR. Therefore, we investigated the effect of induced and extinguished social fear on physical behavior as well as hypervigilance and attentional avoidance toward virtual female and male agents in HSA and LSA students in the SFC paradigm from Reichenberger et al. (2019), where participants actively approached different agents using a joystick. The outcome variables include fear ratings, physical behavior (duration of approach), hypervigilance (time of the first fixation), and attentional avoidance (count of fixations).

Concerning fear ratings, our results showed that social anxiety was successfully acquired and extinguished, except for a clear SFC in HSA men. It would be interesting to investigate whether this results comes from differences in coping strategies in HSA men, from somehow reduced conditionability, from reduced attention or higher variance between HSA men. The findings on the physical behavior might give a preliminary hint that HSA seemed to need more time to approach all agents during fear acquisition than LSA participants, which could mean that HSA were more carefully in approaching agents in this social interaction indicating enhanced social anxiety. With regard to hypervigilance, the HSA fixated the CS+ agents earlier than the CS− agents at the beginning of the fear acquisition, whereas at the end of the fear acquisition they fixated the CS+ agents later than the CS− agents. On the contrary, in LSA participants initial attention did not distinguish between the CS+ and CS− agents during fear acquisition. Moreover, according to our first hypothesis, we found an increased attentional avoidance to CS+ compared to CS− agents for HSA participants during fear acquisition. In contrast, in LSA participants sustained attention did not differ between the CS+ and CS− agents during acquisition, although they reported higher fear ratings for the CS+ than the CS− agents. Therefore we could conclude that LSA might not distinguish between an aversive or not-aversive human according to measures of attentional avoidance. Regarding fear extinction, the variation in attentional avoidance toward the aversive and non-aversive agents after fear acquisition disappeared for the HSA and LSA group after the extinction.

Relating to the vigilance-avoidance hypothesis, our results indicate that HSA directed especially their initial attention at CS+ than CS− agents at the first half of the fear acquisition, and avoided subsequently the CS+ more than the CS− agents at the second half of the fear acquisition to possibly reduce emotional distress. Our findings are compliant with the assumption that HSA or persons with SAD guide their initial attention to emotionally threatening information and tend to avoid eye contact or threatening stimuli to might reduce anxiety directly (Chen et al., 2002; Wieser et al., 2009b; Singh et al., 2015; Shechner et al., 2017). For clear evidence of a hypervigilance bias, we would have expected that HSA participants should guide their initial attention more toward threatening than non-threatening agents at the second half of the fear acquisition as well. The results of our current study are in line with Mühlberger et al. (2008) as well, who found that HSA participants avoided emotional facial expressions in a virtual fear-relevant situation. However, the authors could not affirm clear results of hypervigilance to threat-relevant stimuli, like angry faces, in VR. Relating to these results e Claudino et al. (2019) call into question in their review the ecological validity of other studies, which indicated hypervigilance regarding emotions. Most research mainly used visual search and dot-probe tasks or measured gaze behavior (e.g., hypervigilance in time periods of 500–1500 ms) when persons looked at images of different faces on a computer display. In contrast, we utilized a SFC paradigm in VR, in which the participant had to approach different agents during fear learning. Furthermore, our non-clinical sample consisted of low and HSA students without a diagnosed SAD. Most of the studies found clear hypervigilant patterns of attention in persons with SAD.

Social anxiety is related with selective attention to social threatening stimuli and self-focused attention to internal cues, which are assumed as maintaining factors of SAD. For example, self-focused attention on internal cues (e.g., negative thoughts, emotions, and body sensations), could impair the performance and even inhibit perceiving positive social feedback which invalidates the false impressions of how others judge them (Perowne and Mansell, 2002; Spurr and Stopa, 2002).

Perowne and Mansell (2002) examined in LSA and HSA individuals their self-focused and selective external attention of non-verbal behaviors in a social-evaluative stress situation. The authors revealed that the HSA related more self-focused attention and perceived a more negative view of their performance than the LSA group. However, the HSA showed no less selective attention than the LSA group. These findings are in line that HSA persons monitor others and as far as they find a hint for a negative evaluation they turn their attention to internal cues (Clark and Wells, 1995; Perowne and Mansell, 2002).

Thus, the question arises how could self-focused attention influence hypervigilance and attentional avoidance in the current study? With regard to the cognitive model of Clark and Wells (1995), social anxiety leads to limited attentional processing of external cues. Moreover, the remaining reduced attentional processing of the external social situation tends to be biased, as ambiguous behaviors are more likely to be interpreted as negative (Clark and Ehlers, 2002). Therefore, HSA participants should focus more to internal than to external cues. This assumption is partly in line with our findings on the hypervigilance that at the first half of the fear acquisition the HSA fixated more early the CS+ than the CS− agents, whereas at the second half of the fear acquisition they fixated the CS+ later than the CS− agents. With regard to the attentional avoidance, we found for the HSA a reduced amount of fixations toward the CS+ than to the CS− agents at the end of the fear acquisition. Our findings partly support the vigilance-avoidance hypothesis that HSA directed especially their initial attention at threat-relevant agents with their negative hints, and at the end of the fear conditioning they avoided subsequently the threat-relevant more than the non-threatening agents to possibly reduce their emotional distress. For a clearer insight of the impact of self-focused attention to hypervigilance and attentional avoidance, the self- and other-focused attention could be measured with the Focus of Attention Questionnaire (Woody, 1996) in future studies.

There are less empirical findings relating the effects of fear conditioning on gaze behavior. Michalska et al. (2017) showed that children hold their gaze more often and longer toward the area of the eyes of a CS+ than a CS−. The authors used two neutral faces as conditioned stimuli (CS+ and CS−). However, we know no empirical study measuring eye-gaze during fear conditioning with an enhanced ecological validity in VR.

Regarding ecological validity, Foulsham et al. (2011) examined whether gaze behavior in walking through the real world differs compared to watching videos of the same real world in a laboratory setting. The authors showed that the gaze behavior toward the path nearby and objects in the distance is remarkably different in the real world compared to viewing the videos. Moreover, participants looked more often to persons that were close to them on the video than in the real world. These findings demonstrate that the generalization of laboratory results to attentional processes in natural situations is restricted. Because, our participants were immersed in a virtual environment, where they could actively move and interact with female and male agents, we can assume different responses than toward photographs or videos of humans (McCall et al., 2016). We assume that behavioral and attentional processes measures in VR is more related to real-life situations than traditional laboratory paradigms. Moreover, an potential key element assessing reliable and valid physical or gaze behavior in a virtual environment is social presence, the feeling of the participant to interact with another sentient being which involve cognitive and emotional processes (Blascovich et al., 2002; Oh et al., 2018; Felnhofer et al., 2019). Thus, future research on attention and emotion regulation should include a measure of social presence in VR.

Furthermore, our study measures gaze behavior during approaching different social agents. Therefore, the question arises how physical behavior is related to gaze behavior. McCall et al. (2016) showed in their Affect Gallery paradigm that participants gazed more and came closer to positive than negative images. In contrast, the authors presented in their Crowded Room paradigm that participants gazed more directly at agents with angry or sad than neutral facial expressions, but revealed a larger interpersonal distance toward angry than neutral or sad agents. These results illustrate that gaze behavior does not have to match continuously with physical behavior. For example, in a social situation attention is more directed to a threat-relevant facial expression causing to physically avoid the potential threatening person.

Weeks et al. (2019) recommend the use of eye-tracking techniques in more future studies as these suitable tools could improve social skills training (e.g., providing an objective index of gaze avoidance) or in maximizing the effectiveness of therapeutic exposures (e.g., measuring and/or preventing the potential role as safety behavior). Furthermore, in order to investigate the mechanisms of physical and gaze behavior as an important feature in emotional learning and maintaining processes of social anxiety, eye-tracking indices are an interesting implicit method within fear conditioning.

Some study limitations should be noted. First, our non-clinical sample of mainly young students should be considered in generalizing the results to an additional context. Thus, to generalize our current findings we need a more diverse sample with different demographic and even clinical characteristics. Second, technical failure in eye-tracking led to high data loss for seven participants, which may have impaired the likelihood of determining higher order interactions. Third, in future studies we should also define AOIs for the eyes and mouth region of the agents to investigate possible effects of emotional facial expression within a virtual social interaction.

Moreover, we want to note that the applied US (sound of spitting attended by an aversive air blast) during fear acquisition might not only signal potential social, but also physical harm and might induce general fear or even disgust. However, Grillon et al. (2004) revealed that the anticipation of an air blast was not aversive enough to induce a fear-potentiated startle reflex by unpredictable aversive events in humans. We think that being spat followed by the verbal rejection is rather a social relevant than a physical relevant US within a social situation. Thus, we assume that the triggered fear in our experiment is more a social fear of negative evaluation than a physical impairment. Furthermore, we did not receive any feedback regarding disgust, but unfortunately we used no standardized ratings or questionnaire of disgust, which should be measured in future studies. Moreover, it might be interesting to develop a measurement instrument to disentangle physical and social fear associated with the spit stimulus.

Furthermore, it is important to note that our power analysis based on the data from our prior study by Reichenberger et al. (2017) that contained two between subject conditions defined by social anxiety (LSA vs. HSA). In our current study, we included one more between subject condition defined by participants’ gender (women vs. men). In detail, although our LSA and HSA group consisted of an equally distributed ratio of gender as mentioned above, the size of each condition is thus smaller. However, our main hypothesis does not relate to complex interaction effects of social anxiety and gender.

In conclusion, the current study demonstrated the advantages of VR in investigating physical and gaze behavior in social interactions in a highly standardized, experimentally controlled and ecological way. Our results show that HSA seemed to be more careful in approaching agents, fixated the CS+ earlier than the CS− agents at the first half of the fear acquisition, and showed increased attentional avoidance to CS+ compared to CS− agents during SFC compared to LSA participants. Further research could contribute to establishing an implicit and objective measure of physical and gaze behavior, which could potentially serve as a biomarker for SAD.

## Data Availability Statement

The datasets generated for this study are available on request to the corresponding author.

## Ethics Statement

The studies involving human participants were reviewed and approved by Ethics Committee of the University of Regensburg. The patients/participants provided their written informed consent to participate in this study.

## Author Contributions

JR: study conception, data acquisition and analysis, and writing the manuscript. MP: data analysis and contribution to the manuscript. AM: study conception, data analysis, and contribution to the manuscript. All authors have approved the final version of the manuscript and its submission.

## Conflict of Interest

AM is stakeholder of a commercial company that develops and sells virtual environment research systems. The remaining authors declare that the research was conducted in the absence of any commercial or financial relationships that could be construed as a potential conflict of interest. The reviewer SB declared a past co-authorship with one of the authors, AM, to the handling Editor.
